# Advances in the application of hydrogel-based scaffolds for tendon repair

**DOI:** 10.1016/j.gendis.2023.04.039

**Published:** 2023-07-07

**Authors:** Renqiang Chen, Fanglin Chen, Kenian Chen, Jian Xu

**Affiliations:** a Department of Orthopedics, The First Affiliated Hospital, Zhejiang University School of Medicine, Hangzhou, Zhejiang 310003, China; b Department of Orthopedics, The Fourth Affiliated Hospital of Guangxi Medical University, Liuzhou, Guangxi 545005, China

**Keywords:** Application, Hydrogel-based, Repair scaffolds, Review, Tendon injury, Tissue engineering

## Abstract

Tendon injuries often lead to joint dysfunction due to the limited self-regeneration capacity of tendons. Repairing tendons is a major challenge for surgeons and imposes a significant financial burden on society. Therefore, there is an urgent need to develop effective strategies for repairing injured tendons. Tendon tissue engineering using hydrogels has emerged as a promising approach that has attracted considerable interest. Hydrogels possess excellent biocompatibility and biodegradability, enabling them to create an extracellular matrix-like growth environment for cells. They can also serve as a carrier for cells or other substances to accelerate tendon repair. In the past decade, numerous studies have made significant progress in the preparation of hydrogel scaffolds for tendon healing. This review aims to provide an overview of recent research on the materials of hydrogel-based scaffolds used for tendon tissue engineering and discusses the delivery systems based on them.

## Introduction

**Figure 1:**
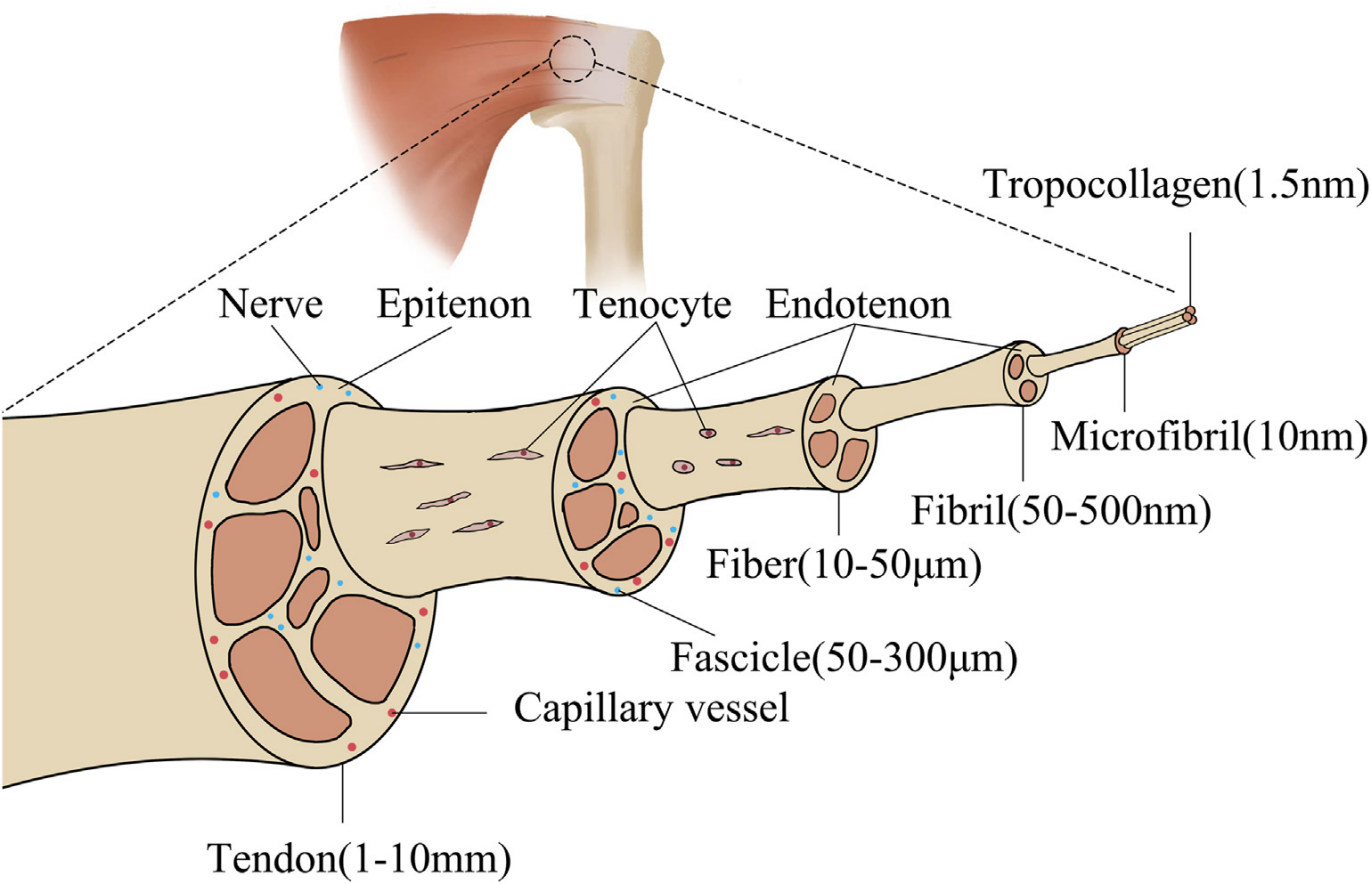
Anatomy of the native tendon.

Tendons play a crucial role in the musculoskeletal system by connecting muscles to bones and transmitting the forces generated by muscle movement to bone tissue.^1^^,^^2^ As a result, tendons are subjected to substantial tensile forces and act as a buffer to avoid stress concentration, making them vulnerable to damage.^3^ The native tendon is formed by the aggregation of aligned tropocollagen (1.5 nm) into increasingly complex structures, such as microfibrils (10 nm), fibrils (50–500 nm), fibers (10–50 μm), fascicles (50–300 μm), and tendons (1–10 mm) successively.^3^, ^4^, ^5^ Fascicles and their inferior units are surrounded by the endotenon,^5^^,^^6^ connecting with the epitenon, which covers the tendon and minimizes friction with a protective membrane (Fig. 1).^7^ Histological sections show that the extracellular matrix (ECM) of tendons is rich in type I collagen and a small number of cells are present.^6^ Tenocytes, the primary cells residing in the fascicles and periphery, synthesize ECM components and regulate the development and maturation of tendons via various signaling pathways, including TGF β-Smad2/3 and ERK1/2.^4^^,^^5^^,^^7^, ^8^, ^9^ More in detail, the TGF β-Smad2/3 signaling pathway can up-regulate scleraxis (Scx), tenomodulin (Tnmd), and other tenogenic markers. Tendon healing occurs naturally via intrinsic and extrinsic mechanisms. Intrinsic healing refers to the proliferation of tendon cells and ECM formation, while extrinsic healing occurs via the invasion of fibroblasts from the surrounding tissues.^8^ Due to the relatively low number of tendon cells and the low activity of growth factors, tendon healing is primarily extrinsic. Furthermore, the poor vascularization of tendons exacerbates their limited intrinsic repair ability after injury.^6^^,^^9^ In the rotator cuff, there are four different zones in the tendon-to-bone interface (TBI): ligament tissue, unmineralized fibrocartilage, mineralized fibrocartilage, and bone tissue, which are difficult to reconstruct.^10^^,^^11^

As fitness exercises become more popular among citizens, tendon injuries (TI) have become increasingly common. Surveys have shown that TI is among the 15 most prevalent diseases in the United States.^12^ Traditional treatment options for TI include conservative treatments and surgical interventions, such as sutures and autografts, which are associated with potential problems.^13^, ^14^, ^15^, ^16^ For example, latissimus dorsi transfer and pectoralis major transfer for rotator cuff tear (RCT) often fail as a result of mismatched mechanical properties and anatomical damage at the surgery site.^13^ The disease transmission and donor site morbidity are associated with tendon grafting.^17^ Difficulties in restoring the structure and function of injured tendons make tendon repair a major challenge in sports medicine today.^18^ Strategies to engineer tendon tissue have gained substantial attention to overcome these shortcomings along with the use of hydrogel-based scaffolds.

Tendon tissue engineering has emerged as a promising approach to regenerating tendon tissue that functions similarly to natural tendons. This involves loading cells or other substances onto scaffolds that can integrate with surrounding tissues.^17^ Scaffolds can create a three-dimensional (3D) environment that resembles ECM to support the proliferation and differentiation of cells.^19^ Hydrogels and synthetic polymers are the most used materials for scaffold fabrication. As extremely hydrophilic and cross-linked network materials, hydrogels possess high water content, elasticity, and bioactivity,^16^^,^^20^^,^^21^ beneficial for the penetration of nutrients and biofactors,^22^ and act as carriers for cells and drugs with minimal immune reactivity.^16^ However, a majority of traditional hydrogels are inferior to synthetic materials in mechanical properties and controllability.^17^ Thus, hydrogel-based scaffolds, containing hydrogels with/without synthetic polymers, are developed in the delivery systems of tendon tissue engineering (Table 1, Table 2).

**Table 1:** Materials used for tendon tissue engineering.

Hydrogels	Advantages	Disadvantages	References
Natural hydrogels			
Gelatin	Sustainable release, simple and economical manufacture ion, and hydrophilicity	Risk of disease transfer and poor mechanical property	17,26,40,54,57
Hyaluronic acid	Viscoelasticity and hydrophilicity	Local reaction, poor mechanical properties, and biocompatibility problems in synthesis	22,29,63,64
Fibrin	Easy availability, low cost, and suitable for tenogenic differentiation	Rapid degradation and poor mechanical properties	48,63,67,68,123
Chitosan	Biocompatibility, adhesive capacity, and anti-peritendinous adhesion	Poor mechanical property and inflammation reaction with highly acetylated chitosan	80,85
Alginate	Injectability, self-healing, and bio-degradability	Must be modified by oxidation to degrade; no intrinsic cell binding parts and poor mechanical property	2,27,88,91
Collagen	Low immunogenicity and bio-degradability	Poor physicochemical plasticity, low denaturing temperature, and fast degradation rate	25,81,93,95,124,125
tHGs	Biocompatibility and specific elements for tendon regeneration	Disease transmission, heavy cost, ethical issues, and poor mechanical property	38,108,109
Synthetic polymers			
Polyglycolic acid	Absorbability and rapid degradation	Relatively poor mechanical property of unwoven polyglycolic acid	11,31,114
Poly lactic-co-glycolic acid	Biodegradability, biocompatibility, and proper mechanical properties	Poor osteoinductivity ability	23,45,118,121
Polycaprolactone	Biodegradability, biocompatibility, and strong mechanical strength	Not conducive to cell adhesion, slow degradation rate, and low biological activity	41,56,110, 111, 112, 113
Poly-l-lactic acid	Biodegradability and biocompatibility	Accumulated acid production by the degradation and obvious inflammation, poor osteoinductivity ability	10,120,121

**Table 2:** The comparison of different delivery cells.

Loaded cells	Strengths	Weaknesses	References
Bone marrow mesenchymal stem cells	Pluripotent potential and great proliferative capacity	Differentiation uncertainty, scarcity, donor-site morbidity, and high cost	31,33,116,129,134,138
Adipose-derived stem cells	Accessible harvest and expansion, abundant availability, and immunomodulatory effect	Differentiation uncertainty and short survival time *in vivo*	31,104,128,129,134
Tendon-derived stem cells	Pluripotent and clonogenic potential and self-renewal	Slow proliferation and mutation	18,130,139,140
Dermal fibroblasts	Easy availability and similarity with tenocytes	Inferior quality of the forming tendon	104,133, 134, 135
Tenocytes	The inherent cells of the tendon and being robust within the tendon	Few sources, losing phenotype at *in vivo* expansion, and donor-site morbidity	104,133,134

Although hydrogel-based scaffolds composed of various materials have been used to regenerate tendons, there is still a need for an ideal biodegradable scaffold that can mimic the architecture of the native tendon ECM. Specifically, except for the necessary properties of non-toxicity and degradation in accordance with the formation of the new tissue, the ideal scaffold should be designed with mechanical strength to stimulate the tenogenic expression of cells, sustain the release of biofactors or drugs while degrading, and have a versatile structure for cell adherence and nutrition exchange. Furthermore, to minimize inflammatory response while withstanding the intense pressure exerted by muscles and joints, it needs to maintain its integrity.

## Properties of scaffolds

### Biocompatibility and biodegradability

As the graft is transplanted into the body, scaffolds ought to possess superior compatibility and degradability.^23^^,^^24^ Synthetic polymers tend to cause foreign body reactions, induce synovitis, and have insufficient biocompatibility,^25^ as compared to natural materials like gelatin^26^ and alginate.^27^ For instance, an implanted sheet of poly-l-lactate-epsilon-caprolactone (PLC) for treating rotator cuff defects, resulted in numerous multinuclear cells around the remaining sheet 16 weeks post-implantation,^11^ indicating a lack of biocompatibility. Therefore, to ensure biocompatibility and degradability, toxic side products should not be produced while manufacturing scaffolds. Scaffolds made from hyaluronic acid (HA) hydrogels, containing methacrylate (HA-ME) fabricated by photopolymerization, are more toxic than catechol-functionalized hyaluronic acid (HA-CA), produced by oxidative cross-linking.^22^^,^^28^ Park et al compared the viability of adipose-derived stem cells (ASCs) cultured in both HA-ME and HA-CA and reported that approximately 93% of ASCs in HA-CA were active during seven days of culture, significantly higher than those in HA-ME (*P* < 0.01).^22^ As scaffolds degrade, they lose their mechanical properties and components. Therefore, the rate of degradation should be in concordance with the regeneration rate of the tendon tissue.^29^

### Mechanical property

It is widely accepted that tendons function within a dynamic environment and bear strong force *in vivo*.^30^ The mechanical properties of a scaffold has a significant impact on the formation and maturation of engineered tendon tissue.^31^ Mechanical stress induces the expression of proteoglycan and versican, which are critical proteins for the niche composition and matrix maturation of tendon-derived stem cells (TDSCs).^32^ Ciardulli et al demonstrated that the expression of Scx in bone marrow mesenchymal stem cells (BMSCs) was up-regulated up to 1600-fold in dynamic conditions (culturing in cyclic strain stimulation) compared with just 800-fold in static conditions, emphasizing the importance of mechanical stimuli in tissue engineering.^33^ Consequently, scaffolds are required to deliver strain or compression inputs to the loaded cells. To fabricate scaffolds with anisotropic mechanics, electrospinning had been successfully attempted.^30^^,^^34^^,^^35^ Previous publications had shown that scaffolds supplemented with bioactive ions showed remarkable promotion in mechanical tests.^24^ Ma et al manufactured composite hydrogels of calcium silicate (CS) nanowires/alginate using the 3D printing method, which significantly increased the tensile strength and Young's modulus of the composite hydrogels.^36^ Additionally, the relation between them was positive.

### Structure

A porous structure with a high surface-to-volume ratio can facilitate the process of cell adherence, proliferation, and differentiation into tenocytes because the properties prompt oxygen and nutrient diffusion in case the vascularization is poor.^19^^,^^37^^,^^38^ Lyophilized tendon-based hydrogels demonstrated superior cell proliferation and platelet-rich plasma (PRP) activation compared to fresh tendon hydrogels due to high porosity and greater exposure of collagen I after lyophilization.^38^ Once the tendon sheath is destroyed, extrinsic fibroblasts invade the injured sites, resulting in peri-tendinous adhesions.^39^ To prevent exogenous fibroblast invasion, scaffolds mimicking the function of the tendon sheath must be equipped with a small pore size to block cell migration and ensure nutrient exchange simultaneously.^7^

To enhance cell adherence, the surfaces of scaffolds should be relatively rough and not too hydrophilic.^10^^,^^29^ The hydrophobic nature of polycaprolactone (PCL) scaffolds leads to insufficient cell attachment and tissue integration.^40^^,^^41^ Lv et al demonstrated that supplementation of hydroxyapatite (HAP) enhanced the roughness and hydrophilicity of the poly-l-lactic acid (PLLA) nanofibrous membrane, improving cell attachment.^10^ Using the technology of electrospinning, composite scaffolds with tendon-like tissues possessing aligned topography were successfully produced,^30^^,^^37^ contributing to the elongation of cell shape and inducing tenogenic differentiation.^10^^,^^30^^,^^37^^,^^42^^,^^43^ Aligned and dense fibers are equipped with mechanical properties resembling that of the native tendons.^44^ Moreover, for scaffolds of aligned fibers, microscaled-aligned topography is more conducive to inducing tenogenic differentiation compared to the nanoscaled topography,^43^ facilitating better cell adhesion and elongation.^45^ Furthermore, studies have shown that aligned scaffolds are instrumental in the modulating inflammatory response, promoting the shift of macrophage subpopulations from M1 to M2.^45^^,^^46^ The pro-inflammatory phenotype of M1 macrophages initiates inflammation while M2 subpopulations play an anti-inflammatory role in the immune system.^45^^,^^47^

### Injectability and self-healing property

**Figure 2:**
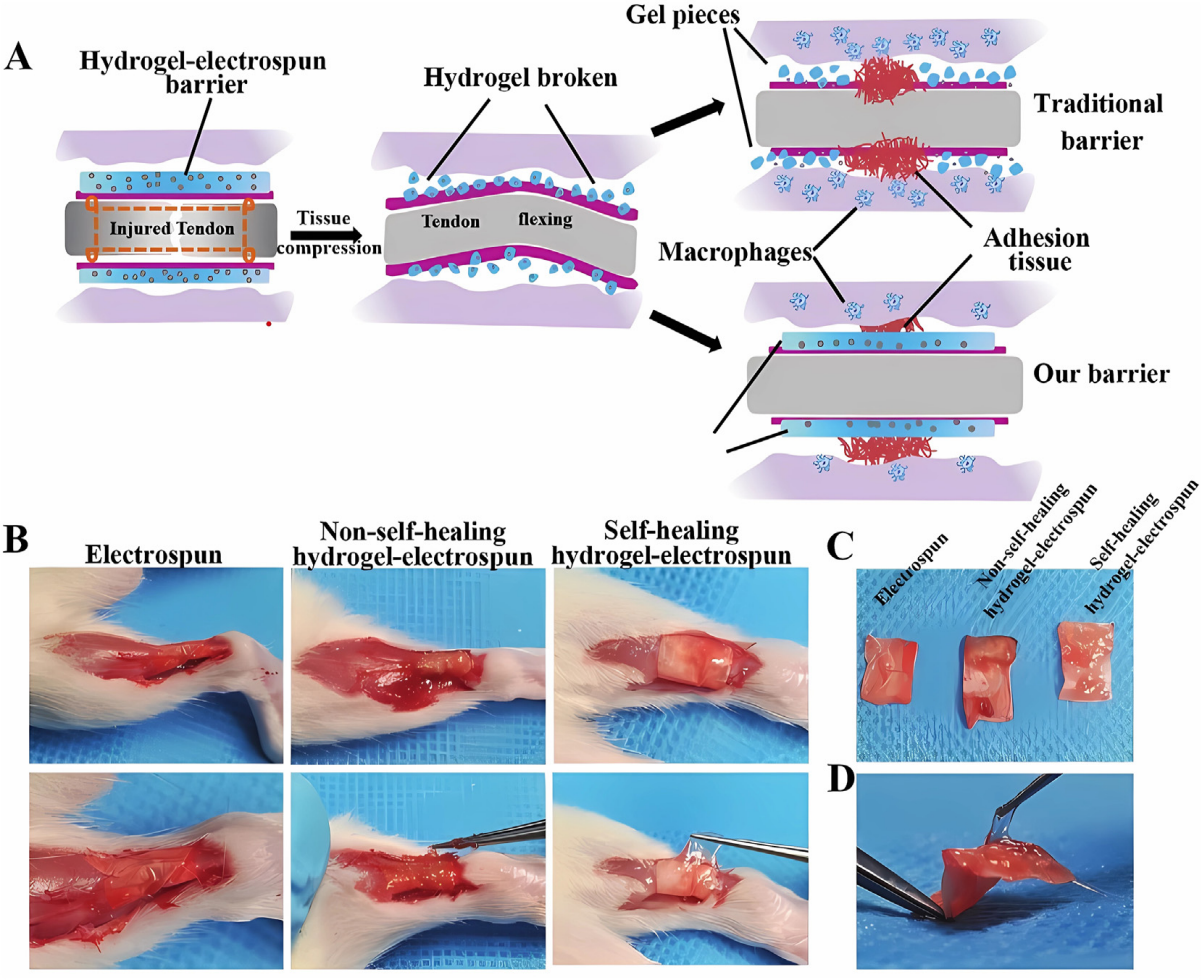
The self-healing property of hydrogel. **(A)** The effect of self-healing hydrogel under tissue compression. The self-healing hydrogel keeps its shape, while the traditional one is broken into pieces, inducing a severe inflammatory reaction. **(B**–**D)** The macroscopic shape of the electrospun nanofibers post-implantation *in vivo*. The self-healing hydrogel attached to the electrospun nanofibers keeps its integrity, but the non-self-healing hydrogel is destroyed. Reprinted with permission.^48^ Copyright 2021, John Wiley and Sons.

Tendons are essential for transferring strength from muscle to bone,^1^ and the force between tendons and peri-tendinous tissues remains remarkably strong,^1^^,^^48^ reminiscent of the possibility of scaffold's destruction. Once broken, the debris can induce an inflammatory reaction, impeding tissue repair. The self-healing property of scaffolds refers to their ability to recover their shape and function through the internal covalent bonds after they are destroyed (Fig. 2). These covalent bonds, such as disulfide, imine, and hydrazine bonds, exist in the crosslinking of functionalized polymers during scaffold fabrication.^48^ Wang et al developed an imine bond-based self-healing scaffold that returned to its original shape after being cut without external stimulation.^49^ Dynamic Schiff base bonds, formed by amino groups and aldehyde groups, also provide scaffolds with favorable self-healing and injectability properties.^24^^,^^50^^,^^51^ Unfortunately, chemical crosslinking with covalent bonds can lead to the production of toxic substances. Double network scaffolds via dual physical crosslinking not only eliminate the use of toxic chemicals but also display visible self-healing behavior.^52^ Injectability is crucial for filling irregularly shaped repair sites, where scaffolds can easily fill the sites appropriately.^24^^,^^48^ Traditionally, the direct injection of cells can cause cell loss due to penetration into surrounding tissues,^14^ or cell death due to the shear stress during the injection.^53^ As a result, incorporating cells, bioactive factors, or drugs into injectable hydrogels is a suitable option for patients who prefer less invasive treatments.

## Material categories of hydrogel base scaffolds

### Natural hydrogels

#### Gelatin

Gelatin, a hydrolyzed product of collagen, possesses favorable characteristics for tissue engineering such as high hydrophilicity, biocompatibility, and low manufacturing cost.^17^^,^^40^^,^^54^ The amino acid sequence of gelatin, Arg-Gly-Asp acid (RGD), enhances cell attachment to the scaffold by interacting with cell integrins.^17^^,^^54^, ^55^, ^56^ Gelatin can take various forms *in vitro*, such as sheets, microspheres, and sponges; however, it undergoes enzymatic degradation *in vivo* to produce a water-soluble fragment that facilitates the sustainable and stable release of drugs or active factors.^26^^,^^57^ Taking advantage of this property, Zhang et al loaded PRP in a porous gelatin sponge (GS).^26^ They found that GS was sufficiently stable to sustain the bioactivity of PRP, with the release kinetics corresponding with the degrading curve. Tokunaga et al used gelatin hydrogel as the carrier of fibroblast growth factor-2 (FGF-2) and observed a persistent release for two weeks with the degradation of gelatin.^58^ The study also discerned repairing the rotator cuff in rabbits with gelatin alone had no promotion effects. Porous and injectable gelatin microcryogels have also been created to enhance cell-to-cell interactions with a biomimetic 3D structure, which provides more surfaces for cell attachment than 2D structures.^56^ Gelatin methacrylate (GelMA), derived from gelatin by photocuring, has lower antigenicity than collagen hydrogels.^59^, ^60^, ^61^ GelMA can be cross-linked rapidly when fabricating and fills the irregular shape better and thus is widely applied in the delivery system in RCT repair.^59^^,^^62^

#### Hyaluronic acid

HA is a natural glycosaminoglycan that is widely present in human connective tissues, synthesized by synoviocytes and ﬁbroblasts.^1^^,^^63^ A study showed that HA enhances TDSC viability and type I collagen expression in a dose-dependent manner, independent of its molecular weight.^64^ Fabrication of HA hydrogel using methacrylate, aldehyde, and other functional groups causes biocompatibility problems, whereas catechol-functionalized HA manufactured by oxidative cross-linking is less toxic.^22^ Cai et al synthesized self-healing HA using oxidative HA, which incorporated aldehyde groups (HA-CHO), and adipic acid dihydrazide-modified HA (HA-ADH).^48^ These modified HAs sustained their self-healing property even after degradation testing due to rebuilding the dynamic covalent bond inside HA. Scaffolds coated with HA effectively inhibit tissue adhesion because of the small pore size and heavy hydrophilicity of HA coating, which hinders cell immigration.^29^ As the carrier, HA hydrogel not only releases drugs sustainably but also shows anti-inflammatory effects.^65^^,^^66^

#### Fibrin

Fibrin is a natural scaffold for human tissue after injury, which plays a vital role in initiating hemostasis and promoting cellular activity.^67^ The raw materials required to form fibrin can be extracted from a patient's plasma, making it reliable and biocompatible.^67^, ^68^, ^69^ In a study comparing fibrin and collagen, tendon progenitor cells cultured in fibrin gels up-regulated the expression of Scx and Tnmd significantly on the 14th day, resulting in a more aligned ECM being produced.^70^ This finding is consistent with a study by Bottagisio et al, who observed higher tenogenic gene expression in fibrin-based scaffold compared to other mediums.^71^ Sustained release of biofactors *in vivo* can be achieved in fibrin-based delivery carriers with proper concentrations of fibrinogen and thrombin as opposed to the explosive effect of PRP.^72^, ^73^, ^74^ Although fibrin has poor mechanical properties, it can be improved through crosslinking protocols.^68^^,^^69^

Platelet-rich fibrin (PRF) is a concentrate of platelets that contains a substantial number of growth factors and has a porous microstructure.^75^ When implanted into the body, PRF can increase the viability of tenocytes and promote the formation of a mineralization gradient in TBI, which plays an important role in TBI healing.^75^^,^^76^ The degree of the tenocyte's proliferation depended on the PRF dose.^77^ PRF derived from bone marrow could further augment the healing of TBI, due to its higher concentration of growth factors and mesenchymal stem cells (MSCs).^78^ Nonetheless, when PRF and tenocytes were cultivated in a system of small diameter well, adverse factors, including adhesive and fibrinolytic factors, were produced as a result of the gelling effect.^79^

#### Chitosan

Structurally similar to HA hydrogels, chitosan is biodegradable and can modulate inflammation and adhesion.^19^^,^^80^, ^81^, ^82^ It had been proven to prevent the adhesion of flexor tendons and decreased the apoptosis of tenocytes by activating the sirtuin 1 signaling pathway.^83^ Incorporating chitosan into scaffolds can promote the chondrogenesis and osteogenesis of MSCs,^81^ which is suitable for the regeneration of RCT with a multi-organization structure. Chen and colleagues fabricated a chitosan scaffold with an asymmetrical structure consisting of a dense membrane to prevent extrinsic fibroblast invasion and a porous sponge for cell growth.^84^ The outcomes suggested that TDSCs loaded in the scaffold expressed high levels of Scx and Tnmd, with low adhesion scores post-implantation. The inflammatory reaction is a significant challenge in the design of biomaterials for tendon repair. Chitosan with high acetylation tends to result in intense inflammation from M1 macrophages, but low-acetylated chitosan is characterized by a large number of M2 macrophages and anti-inflammatory factors.^85^ Due to the natural origin of chitosan, delivery systems modified by chitosan accelerate the adherence of cytokines and cells.^19^^,^^82^^,^^86^^,^^87^

#### Alginate

Alginate is a heteropolysaccharide derived from brown sea algae, possessing unique properties such as injectability, self-healing, and biodegradability.^2^^,^^27^^,^^88^ In the presence of divalent cations, ionic crosslinking between cations and the carboxyl functional group of alginate results in the stable formation of alginate hydrogels.^16^^,^^89^ Alginate microsphere provides a 3D structure that resembles the native ECM, facilitating the encapsulation of both cells and biofactors.^27^^,^^89^^,^^90^ However, there are no intrinsic integrin-binding domains in alginate, which can hinder the adhesion of cells,^91^ and prevent peri-tendinous adhesion if used as the outer layer of scaffolds.^80^ The addition of RGD covalently enhances the interaction between MSCs and alginate, boosting cell attachment and the availability of nutrients or growth factors.^2^^,^^89^^,^^91^^,^^92^ While the mechanical property of alginate is inferior, it can be strengthened by adding bioactive irons.^36^^,^^89^ To note, the degradation of alginate hydrogels is dependent on the oxidation level and does not affect the compatibility of the scaffolds and cell proliferation.^89^

#### Collagen

Collagen is the main component of the ECM and comprises approximately one-third of the total proteins in vertebrate animals.^93^ As a natural biological macromolecule, collagen has low immunogenicity compared to synthetic materials.^17^^,^^94^ However, the denaturing temperature for collagen is approximately 38–45 °C,^95^ making it unsuitable for thermal preconditioning in tissue engineering.^69^^,^^96^ Research has shown that adding keratin improves the thermal stability of type I collagen hydrogels, maintaining their shape at 47 °C for over an hour.^95^ Generally, porcine collagen has a higher denaturation temperature and lower mechanical properties than bovine collagen.^25^^,^^97^ Although collagen molecules can self-assemble into fibrils, the collagen hydrogels that are ultimately extracted have randomized organization and poor mechanical stability.^44^^,^^93^ Combining collagen with polyglycolic acid (PGA) not only improves the mechanical properties of the collagen sponge,^98^ but also reduces its shrinkage, facilitating the diffusion of oxygen and nutrients.^94^ To meet ethical and mechanical requirements, marine collagen scaffolds can also be considered.^99^

Maeda et al proposed a novel method for fabricating a tendon-like collagen hydrogel that simultaneously realizes fiber alignment and crosslinking,^100^ which are typically performed separately in routine procedures. This method imposed mechanical loading on the collagen gel after polymerizing in the presence of genipin, the agent of chemical crosslinking. The aligned dense collagen scaffolds were produced using the gel aspiration-ejection technique,^53^ which quickly densified and realigned the fibril.^44^ However, these technologies fail to form the topography that resembles ECM *in situ*. To address this, Wright et al developed a non-invasive protocol for remotely controlling the topographical cue.^101^ By incorporating iron oxide magnetic nanoparticles into collagen hydrogels and imposing a magnetic field, a fiber-like architecture was observed, and the effect depended on the dose of magnetic nanoparticles.

### Hydrogels acted on tendon extracellular matrix

Scaffolds based on ECM maintain the native morphology and biological features that are required for cellular proliferation.^102^ Though it is stated in the previous context that ECM of tendons is mainly composed of type I collagen,^6^ the hydrogels based on tendon ECM (tHGs) are not equal to collagen hydrogels as the elements of tHGs are tailored to their physiological and functional requirements, leading to tenogenic differentiation.^103^^,^^104^ Ning et al conducted a comparative study between collagen hydrogels and tHGs,^105^ revealing that some inherent ingredients of native tendon ECM were retained in tHGs, including stromal cell-derived factor-1 and fibromodulin, even though the microstructure of both hydrogels was similar. Furthermore, fibroblasts cultured in tHGs expressed higher levels of paxillin protein, an essential factor for cell attachment and proliferation, compared to those grown in the hydrogels made of type I collagen.^106^ Therefore, tHGs have garnered significant attention in the field of tissue engineering in recent years.

The tHGs are structural and functional protein complexes obtained through decellularization that are biocompatible and capable of forming a gel under body temperature, with an internal porous 3D network structure.^38^^,^^103^^,^^107^ A combination of repeated freeze/thaw processes and nuclease treatment is a more effective method of decellularization than chemical decellularization, which can damage the tendon ECM.^105^ Kaizawa and colleagues found that injecting tHGs after suture repair improved the TBI healing biomechanically of chronic rotator cuff injuries significantly better than repair alone,^107^ which aligned with their previous report.^103^ There are many potential mechanisms concerning the therapeutic effects of tHGs. Franklin et al observed that tHGs augmented acute or chronic healing by attracting systemic ASCs.^108^ Moreover, Ning et al showed that tHGs significantly increased the tenogenic differentiation and migration of TDSCs when cultured in tHGs.^105^ Additionally, lyophilized tHGs exhibit a higher capacity to activate cell proliferation and PRP than do the fresh tHGs.^38^ Using human tHGs is always associated with cost and ethical issues,^109^ which need to be addressed in future research.

### Synthetic polymers

Despite availability and biocompatibility, natural hydrogels have poor mechanical properties and degrade rapidly,^17^ which limits their practical applications. In contrast, synthetic polymers possess excellent mechanical properties and can be produced in large quantities, making them an attractive option for clinical use. To achieve optimal clinical outcomes, a combination of natural and synthetic hydrogels is typically utilized.

PCL is an absorbable and biocompatible polymer and is approved by the U.S. Food and Drug Administration (FDA) for clinical use^40^; however, its limitations, including hydrophobicity, slow degradation rate, and poor cell adhesion, suggest that it should be modified.^41^^,^^110^, ^111^, ^112^, ^113^ Blending PCL with natural hydrogels has been proven to improve these properties, such as gelatin^61^ and alginate.^16^ Specifically, the hydrophobicity and cell adherence of the PCL is enhanced because of the hydrophilic groups and the RGD sequence of natural hydrogels.^17^ PCL modified with chitosan demonstrates improved cytocompatibility, reduced rolling leukocytes, and more apparent vascularization after implantation compared to the PCL control group.^112^ In preventing tendon adhesion, PCL nanofibrous membranes prepared via electrospinning can act as a physical barrier to block fibroblast migration from the peri-tendinous tissues.^113^ The PCL nanofibrous membranes fabricated by Domingos et al not only provided mechanical stability but had a small pore size (0.70 μm), while the average diameter of fibroblasts was nearly 10 μm.^7^

PGA is composed of absorbable components and degrades relatively quickly.^11^^,^^94^^,^^114^ A patch graft of PGA was utilized in a retrospective study to treat irreparable RCTs, which contributed to no local inflammation or subacromial adhesions by the one-year follow-up.^114^ Aligned scaffolds of PGA also promote the tenogenic expression of stem cells by inducing cell elongation.^43^^,^^115^ It is generally accepted that the Achilles tendon withstands the largest mechanical loading from the body and the unwoven PGA had relatively poor mechanical properties when compared with that of the Achilles tendon. To improve this, Deng et al fabricated a composite scaffold comprising unwoven PGA fibers as the inter part, wrapped in a net knitted with PGA and polylactide fibers as the outer part, to provide adequate tensile strength for the repair of Achilles tendon defects.^31^ Additionally, in contrast to the unwoven nanofibrous scaffolds, the woven scaffolds accelerate cell proliferation with a larger pore size between nanofibers, promoting nutrient diffusion.^30^ The versatile co-polyester of PGA, poly lactic-co-glycolic acid (PLGA), can be used to fabricate a 3D microstructure that resembles the native tendon ECM,^23^^,^^116^ except for the necessary properties of PGA. Wrapping PLGA nanofibers around tendon defects has been shown to reduce the formation of tendon adhesion.^117^ Previous findings suggested that aligned PLGA microfiber fleece with a smaller parameter (1.27 μm) resulted in higher expression of Tnmd and lower IL-12 to IL-10 ratio, indicative of better tenogenesis property and immune regulation.^45^ The form of PLGA may also potentially affect its degradation. As the PLGA scaffolds loaded with ibuprofen showed a linear release profile in *in vitro* PBS, a fast burst release occurred when it was implanted into *in vivo* environment or *in vitro* serum.^118^ There are some acidic by-products during the degradation of PLGA, for instance, the olic acid and lactic acid,^117^ which are difficult to metabolize rapidly *in vivo* when achieving high concentrations.^119^

Like the other synthetic hydrogels discussed above, poly-l-lactic acid (PLLA) is biodegradable and can be adjusted in molecular weight to vary the degradation time.^10^^,^^120^ An electrospun membrane made of PLLA and gelatin exhibited remarkable cytocompatibility to fibroblasts and enhanced the formation of aligned collagen fibers.^54^ For the TBI repair, osteoinductivity of the scaffolds plays an important role that is insufficient in PLLA unexpectedly.^121^ The PLLA-HAP nanofibrous membrane designed by Lv et al not only induced more osteogenesis differentiation of BMSCs but decreased the hydrophobicity of PLLA by the hydrophilic groups of HAP.^10^ Similarly, PLLA scaffolds coated with chondroitin sulfate and collagen improved the hydrophilicity of PLLA.^42^ Aligned PPLLA-based scaffolds improve drug release by sustaining their release in comparison with random PLLA scaffolds,^122^ correlated with the crystallinity improvement of the polymer scaffold.

## Delivery systems for tendon repair

### Cell-loaded delivery system

Delivering cells to targeted tissue sites through scaffolds offers several advantages: i) it provides a biomimetic microenvironment for the cells, enabling them to function optimally; ii) scaffolds also protect cells from mechanical damage that may occur during the implantation procedure; iii) it facilitates the maintenance of locally high concentration of cells, further enhancing their therapeutic potential. Given these benefits, numerous types of stem cells and fibroblasts have been utilized in this system.

**Figure 3:**
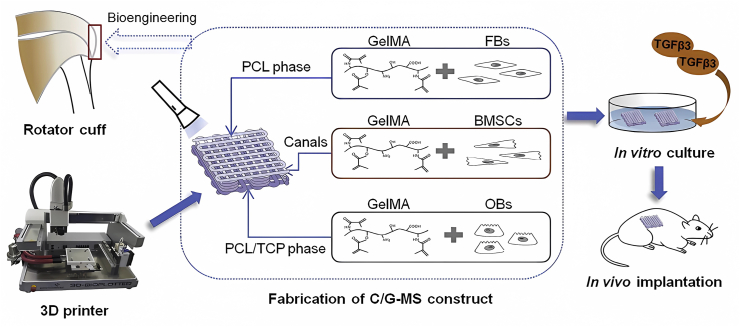
The preparation of multiphasic scaffold. Suspended in GelMA, tendon fibroblasts (FBs) and osteoblasts (OBs) were loaded in the PCL phase and PCL/tricalcium phosphate (TCP) phase respectively, and BMSCs were injected into the ducts between the two phases. Reprinted with permission.^59^ Copyright 2020, Elsevier.

As pluripotent stem cells, BMSCs can differentiate into adipocytes, chondrocytes, and tenocytes under specific conditions.^33^ There are several methods for inducing the tenogenesis of BMSCs, including mechanical stimulation, co-culture with tendon cells, and the utilization of bioactive factors.^126^ An investigation by Bottagisio et al found that fibrin-based scaffolds significantly increased the collagen production of BMSCs, particularly when combined with bone morphogenetic proteins (BMP-14), transforming growth factor β3 (TGF-β3), and vascular endothelial growth factor (VEGF).^71^ In the collagen sponge constructs of Zhang et al, the combination of cyclic stretch and TGF-β1 improved the expression of tendon-relative genes of BMSCs and Young's modulus of the scaffold by decreasing its porosity.^124^ In comparison to tHGs and fibrin scaffolds, BMSCs cultured in demineralized bone matrix exhibited better adherence when exposed to water pressure during arthroscopic repair of RCT.^127^ Additionally, the implantation of BMSCs loaded in PLGA scaffolds, which had a structure similar to native ECM, enhanced the mechanical property of regenerative tissues in rabbit RCT models.^116^ To better meet the structure demand of the native TBI of rotator cuffs, Cao et al invented a multiphasic scaffold through 3D printing that could deliver different cells with PCL and GelMA in a layered manner including tendon fibroblasts, BMSCs, and osteoblasts (Fig. 3).^59^ The cells were simultaneously loaded onto the scaffold and rapidly crosslinked with GelMA to provide better scaffold stability and cell viability.

ASCs, in contrast to BMSCs, are more easily accessible for harvesting and expansion, and they have a reduced osteogenic tendency.^31^ ASCs have been shown to modulate the inflammatory response during the early stage of TI.^128^ By activating the FAK-ERK1/2 and YAP/TAZ signaling pathways, aligned PLLA nanofibrous scaffolds stimulate the immunomodulatory functions of ASCs.^46^ However, the effectiveness of ASCs in promoting RCT healing remains controversial. Kaizawa et al found that there was no significant difference between ASCs-seeded tHGs and tHGs-alone group,^107^ while Kim et al reported that ASCs loaded in fibrin glue decreased the retear rate of RCT after repair.^129^ For Achilles tendon repair, ASCs delivered by porous gelatin microcryogels improved the stiffness and tear resistance of the engineered tissue,^14^ and the ASCs in composite scaffolds of PGA and polylactide fibers showed similar results.^31^

TDSCs are MSCs found in tendons, with self-regenerative and multi-differentiation potential.^130^^,^^131^ Scaffolds made of tHGs induce tenogenic differentiation and suppress the osteogenesis of TDSCs.^132^ The expression of Scx and Tnmd is up-regulated in TDSCs when cultured in tHGs, facilitating their migration movement simultaneously.^105^ Xu et al compared the capability of TDSCs for repairing Achilles tendon rupture in collagen hydrogels and collagen hydrogels enhanced by PRP.^9^ They demonstrated that under the activation of PRP, TDSCs accelerated DNA synthesis and cell proliferation. On the contrary, injecting TDSCs alone had no meaningful treatment effect.^18^ When delivering TDSCs to the TI sites with scaffolds, the seeding density should be considered because the high density of TDSCs enhances the elongated morphology and the expression of tendon-related genes.^131^

Despite their differentiation potential, stem cells may cause ectopic ossification due to the mal-differentiation after implantation. As an alternative, dermal fibroblasts and tenocytes have been used to treat TI. Both originate from the mesoderm and share similarities in morphology and functions for tendon healing.^133^ Dermal fibroblasts using fibrin as the carrier resulted in more regular and denser collagen fibers in the rabbit RCT healing,^133^^,^^134^ better than fibrin loaded with PRP.^134^ Moreover, Chattopadhyay et al indicated that tHGs delivering dermal fibroblasts had a more desirable therapeutic effect for tendon healing than ASCs in the same carrier, owing to the short survival time of ASCs *in vivo*.^104^ Additionally, apart from supporting fibroblast infiltration and viability, researchers also attempted to enhance the mechanical property of scaffolds. Araque et al invented a hybrid scaffold, consisting of a hollow braid of polylactide and microspheres of PLLA and HA inside the braid, coating HA as the outer layer.^29^ The hollow braid of polylactide functioned as the load-bearing component, while the microspheres acted as the fibroblasts carrier, preventing peri-tendinous adhesion with HA coating. Sensini et al fabricated a delivery system based on PLLA and collagen through crosslinking using EDC/NHS reagents to maintain the collagen and mechanical property.^135^ Their results indicated that fibrous morphology was reserved better because of dissolving EDC/NHS in ethanol.

Compared with dermal fibroblasts, tenocytes require extraction from the autologous tendon,^134^ associated with high donor-site morbidity and limited availability, thereby limiting its application. Due to the scarcity of tenocytes, the process of tendon healing tends to be stagnated. To address this, a hybrid interpenetrating system based on alginate hydrogel has been developed, allowing the bidirectional migration of tenocytes, with the seeded tenocytes moving towards the TI sides and the host tenocytes infiltrating the scaffold.^136^ Deepthi et al also designed a biomimetic scaffold in which chitosan-collagen hydrogel was layered on aligned PLLA nanofibers and coated with alginate in the outer layer.^80^ Tenocytes could attach and infiltrate well in this scaffold, and it showed sufficient mechanical property for the flexor tendon healing under immobilization. However, the contraction behavior of tenocytes is prone to destroy the anisotropy of scaffolds and the alignment of tenocytes. Grier et al demonstrated that scaffolds with high crosslinking density and small pore size reduced cell contraction, thus improving the viability of tenocytes.^137^

### Cytokine-loaded delivery system

Cytokines are glycoprotein or peptide molecules that influence cell proliferation and differentiation, for instance, TGF-β, VEGF, basic fibroblast growth factor (bFGF), and platelet-derived growth factor (PDGF). Obviously, the effectiveness of cytokines can be compromised if they are degraded through proteolysis and not delivered to their intended target through a scaffold.^17^ Most cytokines tend to have a short lifespan and are typically unstable *in vivo*,^57^^,^^141^ and as a result, the development of novel strategies to enhance the maintenance and delivery of cytokines has become a key focus in the field of tendon repair engineering.

Incorporating cytokines into scaffolds can be a challenging task, due to the lack of binding domains within the cytokines, despite the intermolecular interactions they form. Researchers have utilized various techniques to effectively incorporate cytokines into scaffolds. For example, acid gelatin has been shown to bind to bFGF through ionic interaction, allowing for the promotion of vascularization and tendon and cartilage regeneration when combined with PRP and bFGF.^142^ In another study, a PCL/gelatin composite was loaded with more VEGF by absorbing a 12 amino acid peptide (DRVQRQTTTVVA) onto its surface.^141^ Heparin is known for its ability not just in anti-coagulation, but the combination with cytokines and other proteins. In the hybrid gelatin/PCL/heparin scaffold, the incorporation of heparin helped to deliver bFGF in a stable manner over a period of 31 days.^17^ This delivery of bFGF prolonged the bioactivity of the protein and improve the gene expression of tenocytes.

**Figure 4:**
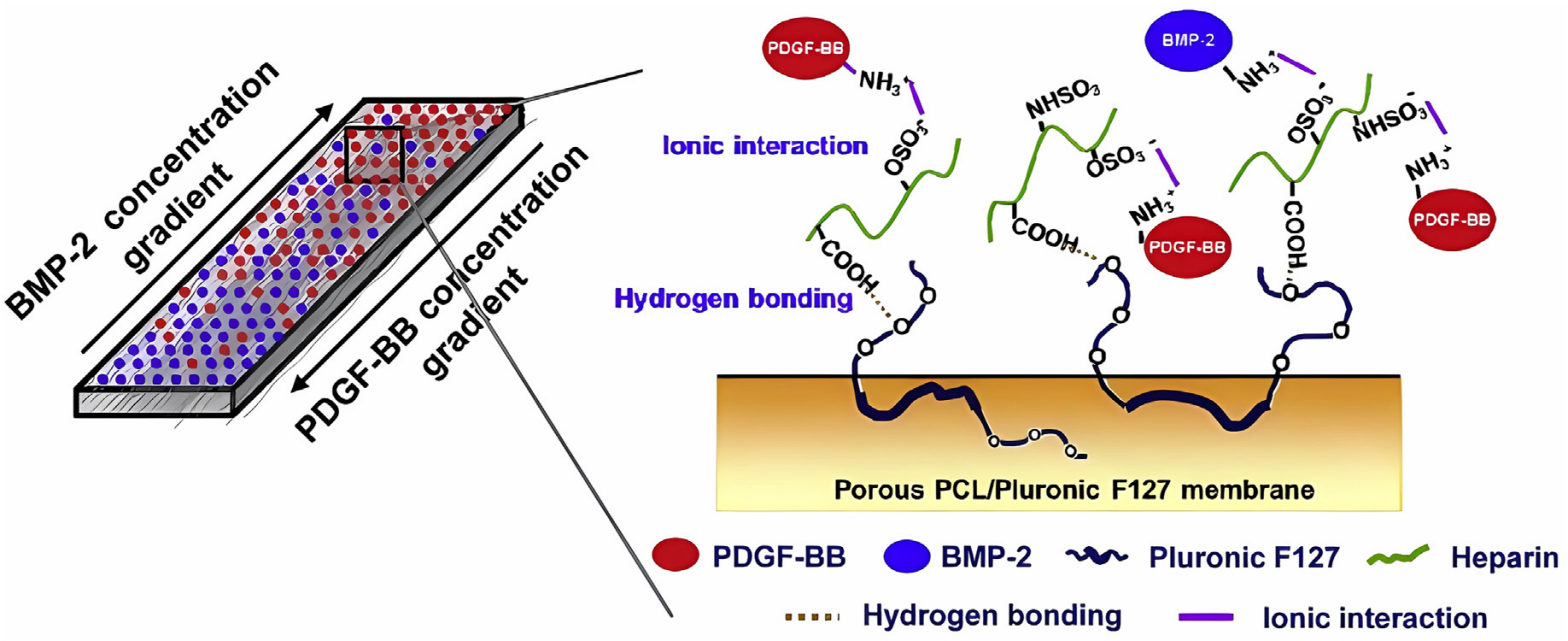
The PCL/PF scaffold with inverse gradients of PDGF-BB and BMP-2. The PDGF-BB and BMP-2 were connected by the hydrogen bonding between heparin and PF, and the ionic interaction between heparin and the two cytokines. Reprinted with permission.^143^ Copyright 2014, Elsevier.

RCT involves inducing cells towards various differentiations, which has prompted the development of delivery systems for multiple bioactive factors. One such system designed by Min et al involved a delivery system using PDGF-BB and BMP-2 distributed in an inverse gradient based on PCL and Pluronic F127 (Fig. 4).^143^ Their *in vitro* analysis showed that a concentration with high levels of PDGF-BB and low levels of BMP-2 induced the tenogenesis of ASCs, while the reverse concentration promoted osteogenic differentiation. Kartogenin (KGN), a small molecule compound, was also utilized to promote the chondrogenic differentiation of MSCs. Researchers found that incorporating it into fibrin glue accelerated the production of fibrocartilage and improved tissue quality in the TBI of rotator cuffs.^73^ Huang et al also loaded KGN into porous GelMA scaffolds that controlled the release of KGN.^62^ Combining this scaffold with bone marrow stimulation resulted in boosted cartilage regeneration in rotator cuff repair post-implantation.

PRP is a platelet aggregate obtained via blood centrifugation, which contains high levels of growth factors.^9^ A meta-analysis by Li et al investigated the clinical outcomes of arthroscopic repair of the rotator cuff with PRP or PRF and showed that the repair group enhanced by PRP had a lower re-tear rate and better pain relief than with PRF.^144^ However, direct injection of PRP into the repairing sites may fail to function in the long term or releases biofactors too quickly. The mRNA of type I collagen in PRP loaded into the gelatin sponge was observed to be higher compared to pure PRP, which is attributed to the sustained stimulation of cytokines.^26^ One study by Xu et al demonstrated that PRP delivered in a collagen scaffold induced maturation of tissue in Achilles tendons, despite the depletion of bioactive factors after three weeks.^9^ The chitosan/PRP construct released more cytokines than PRP clots owing to the activation of chitosan and prolonged the bioactivity of PRP *in vivo*,^87^ resulting in improved radiographic and histological outcomes for the rotator cuff healing of sheep.^82^

### Small molecular inhibitors-loaded delivery system

The formation of adhesive tissues impedes tendon sliding due to the dominance of extrinsic healing and the inflammatory reactions after TI.^39^^,^^84^ Inhibiting the bioactivity of fibroblasts from surrounding tissues or relieving inflammation pharmacologically provides a therapeutic direction for promoting tendon healing. However, the short half-lives of some medications make them unsuitable for treating tendon injuries that require several weeks to heal.^66^ Secondly, the side effects of drugs are multiplied if oral medication is implemented in most cases, such as liver and kidney lesions. While loading medications onto scaffolds enables the consistent delivery of suitable drug concentration to the injury site. Therefore, a basic requirement for the drug carrier is that it should not degrade quickly, which can lead to high drug concentrations that hinder tendon healing.^120^

**Figure 5:**
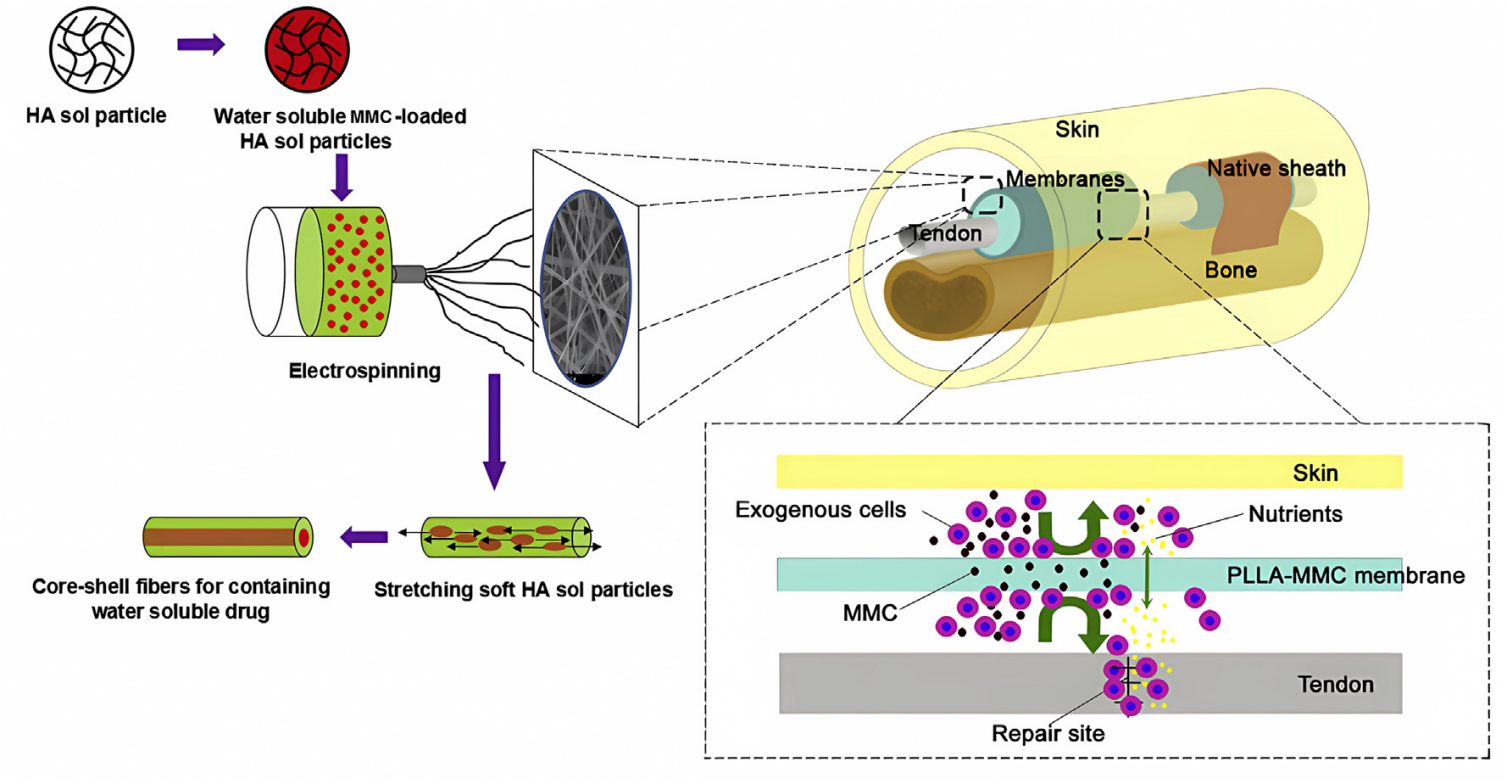
The preparation of PLLA fibrous membranes loading MMC via HA micro-sol electrospinning. The PLLA-MMC membrane acted as the drug carrier and physical barrier to prevent exogenous cell invasion. Reprinted with permission.^120^ Copyright 2015, Elsevier.

Researchers have explored drug delivery methods to restrain extrinsic fibroblasts without affecting intrinsic healing. Mitomycin-C (MMC) is a water-soluble drug that inhibits fibroblast proliferation and collagen synthesis. Zhao et al loaded MMC in the PLLA fibrous membranes through HA micro-sol electrospinning, resulting in the sustained release of MMC in a core–shell structure (Fig. 5).^120^ Results showed that tendon healing was not impaired since only a small amount of MMC was present, suppressing the formation of adhesive tissues. Rhynchophylline (Rhy), a Chinese traditional medication, is also known to prevent adhesion. Nonetheless, Yang et al showed that delivering Rhy with HA hydrogel promoted the expression of tendon-related genes and suppressed the formation of adhesions simultaneously,^66^ though the detailed mechanism was not fully understood.

**Figure 6:**
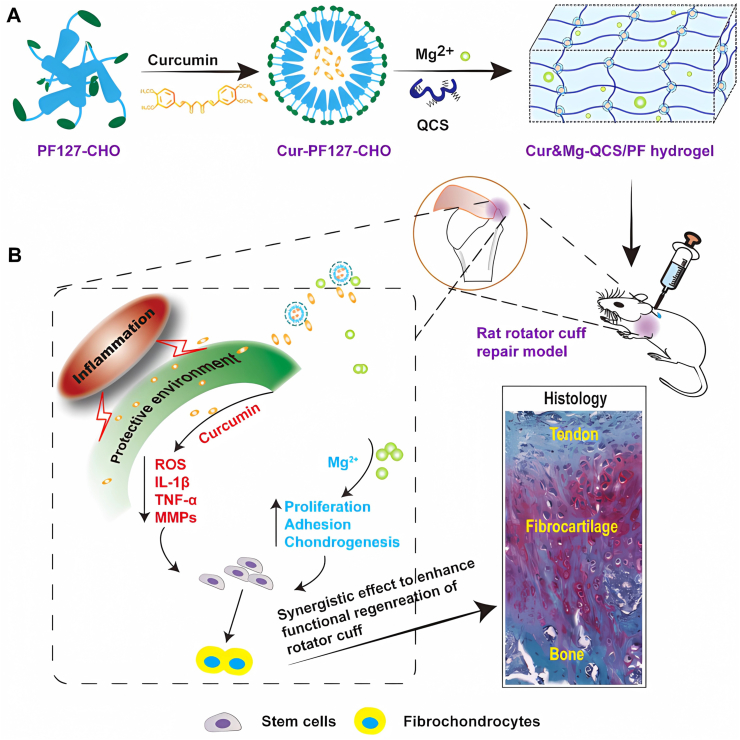
The Cur&Mg-QCS/PF scaffold released curcumin and Mg^2+^ controllably and sustainably. **(A)** The preparation of Cur&Mg-QCS/PF scaffold. **(B)** The therapy mechanism of the scaffold *in vivo*. Reprinted with permission.^24^ Copyright 2021, Theranostics.

Besides, inhibiting oxidative stress and inflammation in tendon repair can also accelerate TI healing. Melatonin (MLT) had been incorporated into PCL/alginate scaffolds to reduce oxidative damage for tenocytes.^16^ *In vitro*, it was conceived that MLT was released faster during the first five days when oxidative damage increased, reducing reactive oxygen species. Although curcumin has great anti-inflammatory efficacy, its oral bioavailability is inferior. For the named Cur&Mg-QCS/PF scaffold, curcumin was released in a controllable and sustainable manner, augmenting the formation of fibrocartilage in TBI with Mg^2+^ synergistically (Fig. 6).^24^ The scaffold was synthesized through the Schiff base bond between quaternized chitosan (QCS) and benzaldehyde-terminated PF. It was injectable and self-healing, and the addition of curcumin and Mg^2+^ resulted in slower degradation of the scaffold. Non-steroidal drugs such as celecoxib could down-regulate the TI inflammation gene expression. Celecoxib loaded in a composite hydrogel of gelatin and Fe_3_O_4_ particles were released further under the magnetic field because of the movement of Fe_3_O_4_ particles, which produced heat and loosened gelatin hydrogel.^12^ Farnesol is a sesquiterpene compound extracted from bacteria and fruits, having an impact on the reduction of inflammatory cytokines and collagen synthesis. A hydrogel membrane composed of gellan gum, HA, and farnesol exhibited slow degradation and rapid swelling, serving as both the physical barrier and farnesol reservoir.^123^ During the RCT repair operation, the malleability of the membrane made it better to fix the sites of TI and improved its clinical effects.

### Bioactive ions-loaded delivery system

The mechanical strength in natural TBI increases gradually from the tendon to bone in view of the four different structures of it, which cannot be achieved with scar formation. While the delivery of cytokines or cells by scaffolds has shown successful tissue regeneration in TBI, there are still some shortcomings, such as rapid diffusing of cytokines,^145^ unstable activity,^57^ and the potential for stem cells to undergo maldifferentiation.^134^ Thus, delivering bioactive ions provides a new strategy to promote TBI healing, as bioactive ions possess multiple potentials in tendon tissue engineering.

HAP, capable of forming a mineral gradient that guarantees the mechanical stimulation required for cell differentiation,^146^ possesses osteoconductivity, and induces osteogenesis in TBI.^147^ Qiu et al achieved a gradient by creating a composite film composed of PCL and HAP with the graded distribution.^146^ They found that the alkaline phosphatase, an osteogenesis symbol, of ASCs in the scaffold exhibited a graded expression in accordance with the content of HAP at day 14, similar to the outcome seen with HAP loaded in PLLA.^10^ Yea et al also created a density gradient of HAP in the ECM scaffold from adipose tissues,^148^ showing that the MSCs seeding on it synthesized specific matrices with respect to the distribution of HAP. However, studies have suggested that HAP is inferior to calcium phosphate silicate materials in terms of osteogenic activity and TBI repair.^149^^,^^150^

Copper ions are important for osteo-stimulation and antibacterial properties, as well as improved vascularization and expression of VEGF.^151^ Scaffold incorporation of copper ions can lead to the regeneration of cartilage and polarization of macrophages to the M2 phenotype.^152^ Zinc ions can also promote the proliferation of tendon tissue via TGF β-Smad2/3 signaling pathway, except for the favorable antibacterial property.^152^ Yang et al designed a bimetallic ion hydrogel with zinc and copper ions by crosslinking with the sulfhydryl groups of gelatin, achieving a sustainable release of the metal ions.^152^ Under the effect of different gradients of the two ions, the fibrocartilage penetration in TBI insertion and the regeneration of bone and tendon was augmented synchronously. As a critical element inside the body, magnesium affects the metabolism of both bone and fibrocartilage in TBI,^24^^,^^153^ involving over 300 enzymatic reactions.^153^ Magnesium ions can induce the osteogenic expression of stem cells, and accelerate the integration between tendon graft and bone.^154^ High concentration of magnesium ions suppresses the ECM calcification.^155^ which is responsible for the tendonitis after TI. It has been demonstrated in the upper context that the stable release of magnesium ions was attained when delivered by Mg-QCS/PF scaffold, boosting the adhesion and accumulation of BMSCs.^24^

## Summary and outlook

Tendon injuries are common problems, and the current pharmacological and surgical treatments for promoting tendon regeneration have proven to be ineffective. Fortunately, the rapid development of tendon tissue engineering is leading to new treatment options. This manuscript reviews the advances of hydrogel-based scaffolds for repairing TI, including their properties, material categories, and delivery system. Natural hydrogels are considered to have excellent immunogenicity and biodegradability; however, they are often limited by their poor mechanical properties, which are complementary to synthetic materials, making hydrogel-based scaffolds a dominant trend. Scaffolds encapsulating cells, cytokines, and drugs offer a promising approach to improving the tendon microenvironment in a way that increases tenogenic expression or prevents adhesion. The repair mechanisms involve several signaling pathways, such as TGF β-Smad2/3 and ERK1/2 that regulate cell differentiation and inflammatory reactions. The MSC delivery to targeted tissues is attached to extensive attention to their pluripotent differentiation, which is further facilitated by cytokines or mechanical stimulation. Nonetheless, whether the treatment outcomes are ascribed to MSCs loaded in scaffolds remains uncertain, as Franklin et al indicated that homing the host stem cells to the repair sites was a mechanism for the effect of tHGs.^108^ Thus, the distribution of MSCs after being transplanted into injury sites and the homing effects on the host stem cells of various scaffolds need to be explored. The application of MSCs also poses challenges such as phenotype drift during preparation and ectopic ossification.^134^^,^^156^ Exosomes, used for cell-free therapy, are focused increasingly because of their biocompatibility, low immunogenicity, and similarity with MSCs in functions.^157^ The delivery of exosomes with hydrogel scaffolds prolongs their retention and releases them sustainably after transplantation,^49^^,^^158^ but loading them into scaffolds to treat TI is relatively uncommon. Several challenges associated with exosomes require further exploration: i) the comparison in therapeutic effect between exosomes from different MSCs when loaded in scaffolds and between exosomes and MSCs,^156^ ii) strategies to improve the endocytosis by the targeted cells after delivering exosomes to TI sides,^49^ iii) the efficacy of diverse hydrogel scaffolds in the delivery of exosomes.

Conditions for successful tendon regeneration are complicated, including vascularization to provide nutrients, mechanical support, and suitable fibrosis without adhesion. Additionally, there are four different tissues in the rotator cuff, necessitating the regeneration of multiple tissues in its repair. The ideal microenvironment of tendon cells requires not only the proper biochemical stimulation but also tendon-like structures to induce tenogenic differentiation. Therefore, these conditions should be taken into consideration when designing the structures and functions of scaffolds in fabrication. For better TBI healing, some strategies have been attempted, including multiphasic scaffold delivering diverse cells,^59^ scaffold equipped with inverse gradients of growth factors,^143^ and scaffold with a gradient of bioactive ions.^159^ Of these strategies, scaffolds delivering bioactive ions with gradient are more attractive, though comparison studies among them have not been accomplished. For one thing, the promotion of both tenogenesis and osteogenesis is acquired in scaffolds of bioactive ions. Their mechanical property caused by the density of bioactive ions, for another thing, stimulates different cell phenotypes in a controllable manner and enables a smooth transition of mechanical strength between the bone and the repaired tendons with the newly formed hierarchical structures.^159^

Self-healing hydrogels can restore the original shape and function even after being broken, making them excellent for preventing peri-tendinous adhesions resulting from the destruction of the synovial sheath of tendons. They can act as a physical barrier to block the invasion of fibroblasts during extrinsic healing or as a membrane to create a smooth surface that reduces friction during tendon movement.^7^^,^^48^ The injectability of hydrogels makes them advantageous for filling irregularly repaired parts with minimal invasion and makes them suitable for use as carriers of drugs or cytokines.^24^ However, the considerable force between tendon and bone can disrupt the integrity of hydrogels, leading to the rapid diffusion of drugs or cytokines.^48^ With the popularity of enhanced recovery after surgery, patients are required to finish early movement after operation. Therefore, hydrogels used for repairing TI in the future should take the self-healing property into account when designing the parameter of hydrogels. The combination of self-healing and injectability of hydrogel realizes the sustainable release of substances and certainly maximizes their clinical effects of them. Functionalizing hydrogels with self-healing properties involves the use of covalent bonds, including disulfide, imine, and hydrazine bonds.^48^^,^^52^ However, toxic byproducts are produced during the crosslinking process, and measures must be taken to eliminate these toxic chemicals, such as changing the crosslinking agents,^22^ or replacing them with physical cross-linking methods.^52^ With respect to carriers, hydrogels in the form of microspheres improve the loading property and culturing microenvironment, which prevents damage to cells when implanted.^29^ Some binding sequences, such as RGD or pDA immobilization, can also be used to modify scaffolds to enhance their loading efficacy, facilitating the adhesion of cells to scaffolds.^17^^,^^160^

Taken together, hydrogel-based scaffolds show promise as an alternative approach for accelerating tendon healing. The selection of hydrogel materials should be based on the specific characteristics of tendons and the feasibility of delivering targeted substances at high local concentrations and sustainable existence. To regenerate TBI, it is necessary to satisfy the demands for synchronous regeneration of different tissues, from mechanical stimulation to nutrition supply. While many hydrogel materials have shown potential as cell carriers, it is still unclear which materials are the most effective for repairing TI. Some experiments have only been conducted only in animal models, so their efficacy and safety in humans need to be further investigated. Tissue adhesion can hinder the sliding of tendons, and future research should aim to combine scaffolds acting as barriers with cytokines and drugs to prevent adhesion formation comprehensively.

## Author contributions

RC and JX conceived the idea and wrote the manuscript. FC and KC searched and selected the qualified articles. JX revised the manuscript. All authors contributed to the article and approved the submitted version.

## Conflict of interests

The authors declare that the research was conducted in the absence of any commercial or financial relationships that could be construed as a potential conflict of interest.

## Funding

This study was supported by the 10.13039/501100004607Guangxi Natural Science Foundation (No. 2020GXNSFBA297089), Youth and Talent Research Project of Guangxi Science and Technology (China) (No. AD21220065), 10.13039/501100001809National Natural Science Foundation of China (No. 82102632 and 82160412), and 10.13039/501100018528Liuzhou Science and Technology Project (No. 2021CBB0106 and 2021CBB0108).
